# Pharmacogenomic predictors of anthracycline-induced cardiotoxicity in breast cancer patients: a systematic review and meta-analysis

**DOI:** 10.1186/s40959-026-00449-3

**Published:** 2026-03-03

**Authors:** Vani Mahathi Bulusu, Bhushan Shah, Sheela Sawant, Akansha Choudhary, Saikat Das, Suruchi Jain, Surulivel Rajan Mallayasamy, Karthik S. Udupa, Neha Arya, Jitendra Singh, Mahadev Rao, Rupinder Kaur Kanwar, Amit Kumar, Murali Munisamy

**Affiliations:** 1 Department of Translational Medicine, All India Institute of Medical Sciences, Saket Nagar, Bhopal, 462020 Madhya Pradesh India; 2 Department of Cardiology, All India Institute of Medical Sciences, Saket Nagar, Bhopal, 462020 Madhya Pradesh India; 3 Department of General Medicine, Tata Memorial Hospital, Parel, Mumbai, 400012 Maharashtra India; 4 Department of Medical Oncology, All India Institute of Medical Sciences, Saket Nagar, Bhopal, 462020 Madhya Pradesh India; 5 Department of Radiation Oncology, All India Institute of Medical Sciences, Saket Nagar, Bhopal, 462020 Madhya Pradesh India; 6 Department of Nuclear Medicine, All India Institute of Medical Sciences, Saket Nagar, Bhopal, 462020 Madhya Pradesh India; 7 Department of Pharmacy Practice, Manipal College of Pharmaceutical Sciences, MAHE, Manipal, Karnataka 576104 India; 8 Department of Medical Oncology, Kasturba Medical College, MAHE, Manipal, 576104 Karnataka India; 9 Department of Laboratory Medicine, Rajendra Institute of Medical Sciences, Ranchi, 834009 Jharkhand India

**Keywords:** Anthracyclines, Breast neoplasms, Genetic polymorphisms, Anthracycline-induced cardiotoxicity, Breast cancer, Pharmacogenomics

## Abstract

**Background:**

Anthracyclines significantly improve overall survival and play a vital role in the treatment of breast cancer. However, they are associated with cardiac dysfunction, which is often irreversible. Although anthracycline-induced cardiotoxicity (AIC) is dose-dependent, the difference in susceptibility patterns suggests the role of pharmacogenomics. Several studies explored the role of genetic variants in AIC. Integrating pharmacogenomic testing with routine anthracycline surveillance will help to predict the individuals who are at risk of developing AIC. Therefore, this current systematic review aims to evaluate and synthesize the evidence on the pharmacogenomics association of cardiotoxicity in individuals receiving anthracyclines for breast cancer treatment.

**Methods:**

PubMed, Embase, and Scopus databases are systematically searched for the literature. After the initial search, 842 records have been identified. Following screening, 18 studies investigating genetic associations with AIC in breast cancer patients were found to be eligible for inclusion in the study. The quality of studies is assessed with the Q-Genie tool. The data is extracted and summarized with odds ratios and corresponding confidence intervals were reported.

**Results:**

A total of 18 candidate gene association studies involving 4,703 breast cancer patients were included in the qualitative synthesis. Out of 57 genetic variants reported, 18 genetic variants (31.5%) are associated with an increased risk, while 3 genetic variants (5.3%) have demonstrated a risk-reducing tendency.

**Discussion:**

Characterizing genetic variants in biological pathways of anthracyclines could inform precision therapy and the development of targeted interventions. The limitations of the synthesized evidence include the inadequate sample size, methodological bias within the included studies, inconsistent findings from different studies, and imprecise effect estimates.

**Conclusion:**

The genetic variants influence susceptibility to AIC in breast cancer patients. Further large-scale studies with longer follow-up are warranted to validate these associations and facilitate their translation into clinical practice.

**Supplementary Information:**

The online version contains supplementary material available at 10.1186/s40959-026-00449-3.

## Introduction

Breast cancer remains the second most diagnosed cancer across the world, with roughly 2.3 million new cases every year. In India, it affects approximately 0.2 million individuals annually, constituting 26.6% of cancers among females and 13.6% across both sexes [1]. Anthracyclines were introduced into clinical practice for breast cancer treatment following the NSABP B-11 trial [2]. Since then, anthracycline-based regimens have improved overall survival (OS) in breast cancer patients. A pooled analysis demonstrated that the anthracycline reduces the recurrence risk of breast cancer by one-third in 10 years and mortality by 20–25% [3].

However, they are among the most frequently used cytotoxic drugs that are responsible for anthracycline-induced cardiotoxicity (AIC) [4]. Long-term follow-up data of breast cancer patients revealed that cardiovascular complications contribute to about 15.9% of deaths in breast cancer survivors [5]. A pooled analysis by Smith et al. reported that anthracycline-based regimens increase the risk of clinical cardiotoxicity by about five times and subclinical cardiotoxicity by about six times compared to a non-anthracycline regimen [6]. The cumulative administered dose of anthracyclines is a key determinant of type 1 cardiotoxicity [7]. For instance, about 28% of survivors who received a combined dose of anthracyclines of more than 300 mg/m^2^ developed signs of AIC, compared to about 7% of survivors who received a total dose of less than 250 mg/m^2^ [8–10]. In addition to cumulative dose, clinical risk factors such as hypertension, diabetes mellitus, and obesity increase the susceptibility to AIC [11].

In the clinical settings, it is also observed that some patients experience AIC despite receiving similar anthracycline doses and having comparable baseline clinical factors, indicating possible genetic predisposition [12]. Several candidate genes have been identified with genetic variants involved in anthracycline transport, metabolism, oxidative stress, DNA repair, cardiac signalling, and autophagy-related pathways, which are associated with AIC in breast cancer patients. The variants in genes such as *ABC*, *SLC*, *CBR*, *UGT*, *GST*, *NCF4*, *NOS3*, *NADPH*, *RAC*, *ETFB*, *TRPC6*, *HFE*, *TP53*, *HLA*, *RAAS*, and *ATG* could be the cause of variations in patients’ cardiac tolerance to anthracycline chemotherapy [13–21]. Several predictive tools are also being developed for the early detection of cardiotoxicity due to interindividual variability [22, 23]. Current methods for assessing cardiotoxicity primarily depend on functional parameters, which may not identify early myocardial injury [24]. Combining genetic predisposition with cardiac monitoring may help identify at-risk patients earlier, enabling timely intervention. Therefore, we aim to systematically evaluate and synthesize the evidence for genetic association and AIC in the breast cancer cohort.

## Methods

### Search strategy

The present systematic review was carried out following the PRISMA guidelines (Preferred Reporting Items for Systematic Reviews and Meta-Analyses) [25]. The protocol has been registered in PROSPERO (registration number: CRD420251040386). A systematic search of PubMed, Embase, and Scopus for articles published in the English language from inception till June 2025 was performed to identify relevant articles assessing the pharmacogenomics association of AIC in breast cancer cohorts. Only peer-reviewed full-text publications were included and grey literature and conference abstracts were excluded. The detailed search strategy is presented in in Supplementary Table 1 (S1) and Supplementary Table 2 (S2).

### Study selection criteria

Two independent reviewers (VMB) and (AC) performed a literature search, title and abstract screening, identification, and selection based on the inclusion and exclusion criteria. Any disagreements were resolved by an independent reviewer (MM). Inclusion criteria were prospective or retrospective human studies in which breast cancer patients received anthracyclines. The studies that have evaluated the effect of genetic polymorphisms and AIC reported clinical or subclinical cardiotoxicity outcomes. The excluded studies include case series, case reports, letters, editorials, commentaries, animal studies, non-English literature studies, review articles, cell line studies, abstracts, summaries, conference reports, studies conducted in non-breast cancer patients, studies in which anthracyclines were not the primary chemotherapeutic drugs, and studies that did not report pharmacogenomic associations with anthracycline-induced cardiotoxicity.

**Fig. 1:**
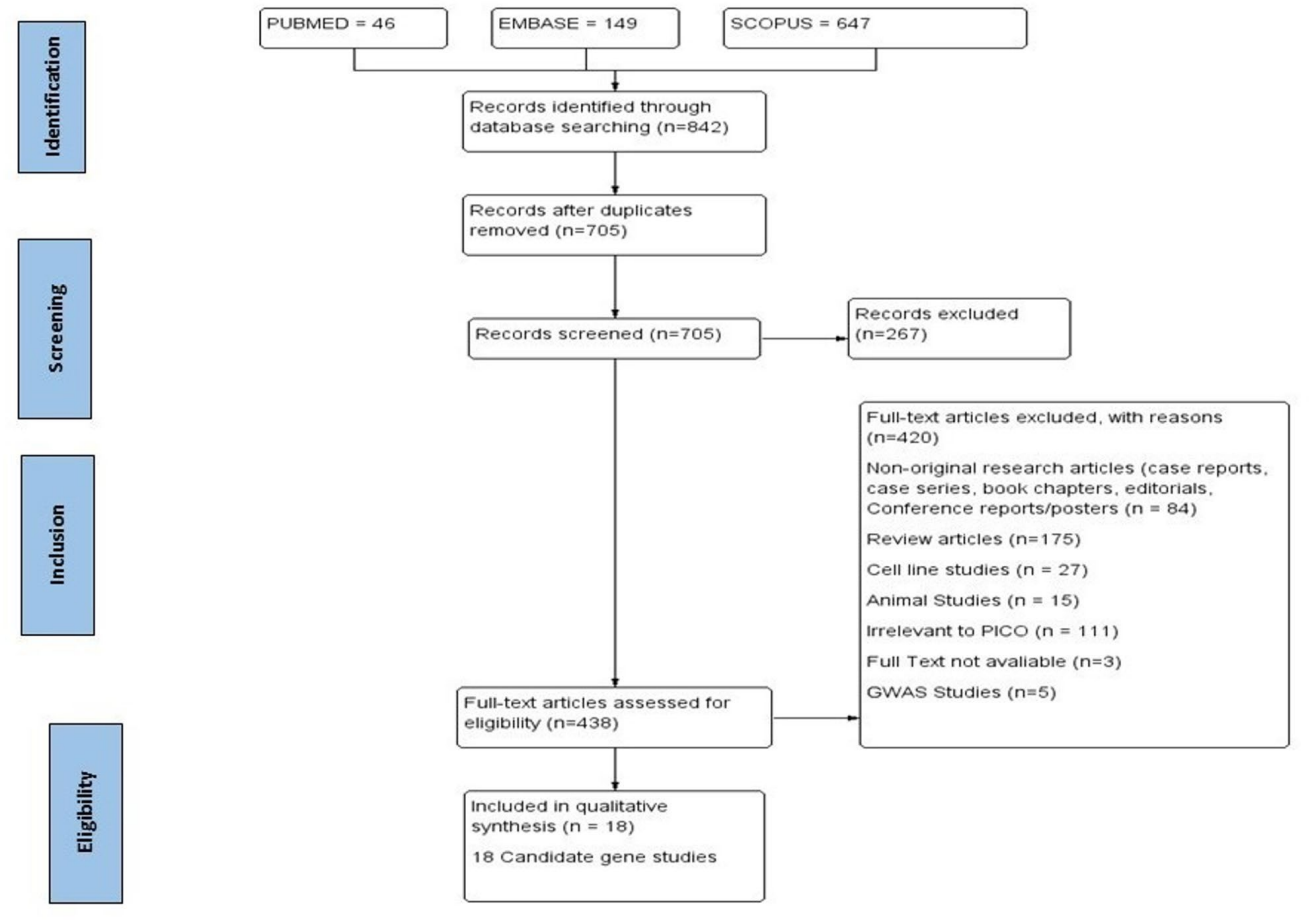
PRISMA Flow chart (Created with RevMan Version 5.4)

A total of 46, 149, and 647 articles were identified from PubMed, Embase, and Scopus, respectively. Following deduplication, 137 articles were excluded. A total of 438 full-text articles were available after screening titles and abstracts. Out of which, 420 articles were excluded for the following reasons: Case reports, Case series, Book chapters, Editorials, Conference reports/Posters = 84, Review articles = 175, Cell Line studies = 27, Animal Studies = 15, Irrelevant to PICO = 111, Full text not available = 3, GWAS Studies = 5 Finally,18 studies are included for qualitative synthesis. The detailed selection criteria of studies are represented in Fig. 1.

### Data extraction

Two reviewers (VMB) and (AC) extracted the data independently from the 18 included studies. Data discrepancies were solved by MM. For all selected articles, the first author, publication year, total sample size, study site, study design, participant characteristics, cumulative anthracycline dose, method of genotyping, cardiotoxicity endpoints, and pharmacogenomics assessed in the study were extracted. We reported the definitions of cardiotoxicity as provided by the authors, which were based on the standards applied in each study. We extracted odds ratios when reported, and we prioritised adjusted odds ratios over unadjusted estimates when they were available. An illustration of the PRISMA flow chart was created using RevMan [26].

### Quality assessment

The selected studies are evaluated for methodological quality by two independent reviewers (VMB), (MM) using the quality of genetic association studies (Q-Genie) tool [27]. Overall, 11 studies were rated as good quality [13–19, 24, 28–30], and 8 were rated as moderate quality [20, 31–36], with no studies categorized as poor. Common strengths of the studies include a clear biological rationale, mentioning the plausible mechanisms for AIC. Technical classification of exposure and outcome measures has been well reported across the studies. Some studies are limited in the categories of sample size [33, 36] statistical analyses [16, 36] and indirectness in the measurement of outcome [35] The individual risk of bias assessment for the included studies is summarized in Table 1.

**Table 1:** Risk of bias assessment

S.no	Study author, Year	Q1	Q2	Q3	Q4	Q5	Q6	Q7	Q8	Q9	Q10	Q11	Overall Quality
1	Advani et al. (2023) [17]	6	5	-	7	4	5	5	6	4	3	6	51 (Good)
2	Domas Vaitiekus et al. (2025) [14]	5	6	5	6	4	4	3	4	5	3	3	48 (Good)
3	Ebaid N et al. (2024) [32]	5	5	4	4	3	3	4	3	4	4	3	42 (Moderate)
4	Gintare Muckiene et al. (2023) [13]	5	6	5	6	5	4	3	4	5	4	3	50 (Good)
5	Grakova et al. (2021) [31]	5	5	4	5	3	3	4	3	4	3	2	41 (Moderate)
6	Hertz et al. (2016) [24]	6	5	5	6	4	5	4	5	5	4	2	51 (Good)
7	Kopeva et al. (2022) [20]	5	5	5	5	3	3	5	3	4	4	2	44 (Moderate)
8	Lang et al. (2021) [15]	6	6	-	6	5	4	4	5	6	4	3	49 (Good)
9	Li et al. (2019) [30]	5	5	4	6	5	4	4	5	6	4	3	51 (Good)
10	Liu et al. (2018) [16]	5	5	4	5	4	5	4	3	4	3	4	46 (Good)
11	Ruiz-Pinto et al. (2018) [29]	5	5	-	5	5	4	4	5	6	3	3	45 (Good)
12	Nyangwara V et al. (2022) [33]	5	5	5	3	4	4	2	4	3	3	3	41 (Moderate)
13	Norton et al. (2020) [19]	6	5	5	7	4	5	5	6	4	3	6	56 (Good)
14	Todorova et al. (2017) [36]	5	5	4	5	4	3	2	3	4	3	4	42 (Moderate)
15	Vaitiekus et al. (2021) [18]	5	6	5	6	4	4	3	4	5	3	3	48 (Good)
16	Vivenza et al. (2013) [35]	4	5	4	5	3	4	4	3	4	3	2	41 (Moderate)
17	Volkan Salanci et al. [34]	5	5	4	5	3	3	4	3	4	3	2	41 (Moderate)
18	Vulsteke et al. (2015) [28]	6	6	5	6	5	4	4	5	6	4	4	55 (Good)

Domain legend

Question 1: Adequacy of the presented hypothesis and rationale

Question 2: Classification of the outcome (e.g. disease status or quantitative trait)

Question 3: Description of comparison groups (e.g. cases and controls)

Question 4: Technical classification of the exposure (i.e., the genetic variant)

Question 5: Non-technical classification of the exposure (i.e. the genetic variant)

Question 6: Disclosure and discussion of sources of bias

Question 7: Adequate power of the study

Question 8: Description of planned analyses

Question 9: Statistical methods

Question 10: Description and test of all assumptions and inferences

Question 11: Conclusions drawn by the authors were supported by the results and appropriate methods

The following cut-points to designate low, moderate, and high quality studies : For studies with case/control status as the outcome of interest: scores ≤ 35 on the Q-Genie tool indicate poor quality studies, > 35 and ≤ 45 indicate studies of moderate quality, and > 45 indicate good quality studies similarly, cut-points for studies without control groups were created by excluding question 3 from the calculation of the total score on Q-Genie: scores ≤ 32 on the Q-Genie tool indicate poor quality studies, > 32 and ≤ 40 indicate studies of moderate quality, and > 40 indicate good quality studies

## Results

A total of 4,703 breast cancer patients were involved in 18 candidate gene studies reporting the association of 57 genetic variants with AIC. The qualitative characteristics of the included studies are presented in Table 2. Out of 57 genetic variants, the odds ratios and corresponding confidence intervals as effect estimates have been reported for 31 genes. Out of which, 18 genetic variants (31.5%) are associated with increased risk, while 3 genetic variants (10%) are associated with a protective effect. The associations between genetic variants and the risk of anthracycline-induced cardiotoxicity, expressed as odds ratios (ORs), are summarized in Table 3. Protective variants are presented in Table 4.

**Table 2:** Qualitative study characteristics of the included studies

S.no	Study: author (year)	Study site	Study design, number of participants	Age (in yrs)	Anthracycline dose used/cumulative dose	Method used for genotyping	Definition of cardiotoxicity	Genes studied
1.	Advani et al. (2023). [17]	Multicentre clinical trial, USA.	*N* = 993, Retrospective replication study within NSABP B-31 RCT cohort	49.6 ± 9.9 years	Doxorubicin 60 mg/m² for 4 cycles cumulative dose:240 mg/m²	MALDI-TOF mass spectrometry	Symptomatic congestive heart failure confirmed by MUGA or echocardiogram, or Probable/definite cardiac death (CD).	*TRPC6* (rs77679196), *BRINP1* (rs62568637), *LDB2* (rs55756123), *RAB22A* (rs707557), *LINC01060* (rs7698718), *CBR3* (rs1056892).
2.	Domas Vaitiekus et al. (2025 [14]	Lithuanian University of Health Sciences (LUHSH) Kaunas Clinics, Europe.	*N* = 81, Prospective study	54.11 ± 9.4 Cases: 54.8 ± 8.9 Controls: 52.9 ± 10.3	Doxorubicin median dose 129.000–303.200 mg/m²	TaqMan genotyping	LVEF Decline of ≥ 10% from baseline to a value ≤ 55% in the absence of clinical symptoms or signs	*SULT2B1* (rs1042637), *UGT1A6* (rs1786378), *CBR1* (rs9024), *CBR3* (rs1056892), *NCF4* (rs1883112), *CYBA* (rs1049255).
3.	Ebaid et al. (2024) [32]	Menoufia University, Egypt.	*N* = 100, Prospective study	45.8 (9.50)	Doxorubicin 60mg/m^2^ for 4 cycles cumulative dose:240mg/m^2^	TaqMan genotyping	Common Terminology Criteria for Adverse Events (CTCAE), ejection fraction was assessed before treat- ment initiation and after 6 months of treatment.	*CBR1* (rs20572), *SLC22A16* (rs714368).
4.	Gintare Muckiene et al. (2023) [13]	Lithuanian University of Health Sciences Kauno Klinikos, Europe.	*N* = 71, Prospective study	53.76 ± 9.23 cases: 53.30 ± 11.25 Controls: 53.94 ± 8.42	Doxorubicin cumulative dose: 236.70 mg/m^2^	TaqMan genotyping	LVEF Decline to a value below the lower limit of normal LVEF < 53%	*ABCB1* (rs1045642), *ABCC1* (rs4148350), *ABCC1* (rs3743527).
5.	Grakova et al. (2021) [31]	Russia.	N 176, Prospective study	45 (42; 47)	Doxorubicin cumulative dose:300–360 mg/m^2^	Polymerase Chain Reaction	LVEF Decline by > 10% at 12 months after chemotherapy and the development of HF with symptoms and clinical signs.	*NADPH* oxidase (rs4673), *NOS3* (rs1799983), *EDNRA* (rs5335).
6.	Hertz et al. (2016). [24]	University of Michigan Comprehensive Cancer Center, USA.	*N* = 166, Cross-sectional study	50 (24–80)	Doxorubicin cumulative dose:240 mg/m^2^ (120–366)	MALDI-TOF mass spectrometry.	LVEF < 55%.	*ABCB1* (rs1045642), *CBR3* (rs1056892), *RAC2* (rs13058338), *NCF4* (rs1883112), *SLC28A3* (rs7853758), *TOP2B* (rs10865801), *UGT1A6* (rs17863783), *CYBA* (rs4673).
7.	Kopeva et al. (2022)[20]	Russia.	*N* = 176, Prospective study	Cases :45 (42; 47), Controls:45 (42; 50)	Doxorubicin cumulative dose: 360 mg/m^2^ (300–360)	Polymerase Chain Reaction	LVEF Decline by > 10% at 12 months after chemotherapy and the development of HF with symptoms and clinical signs	*NOS3* (rs1799983), *EDNRA* (rs5335), *NADPH* oxidase (rs4673), *p53* (rs1042522), *NOS3* (rs1799983), *Caspase 8* (rs3834129, rs1045485), *Interleukin-1b gene* (rs1143634), *TNF-α* (rs1800629), *SOD2* (rs4880), *GPX1* (rs1050450)
8.	Lang et al. (2021). [15]	Roswell Park Comprehensive Care Center, Buffalo, New York, USA.	*N* 155, Prospective study	52 ± 11	Doxorubicin cumulative dose: 240mg/m^2^	TaqMan genotyping	Reduction of LVEF ≥ 5 to < 55% with symptoms of heart failure or Asymptomatic reduction of LVEF ≥ 10 to < 55% defined by CREC.	*CBR3* (rs1056892)
9.	Li et al. (2019). [30]	The Central Hospital of Wuhan, China	*N* = 427, Prospective cohort study.	45.3 ± 6.0	Epirubicin cumulative dose 302.0 mg/m^2^ (281.0–321.0)	Pyrosequencing	LVEF absolute decline of at least 10% from baseline to a value less than 53% on an echocardiogram, Heart failure, acute coronary artery syndrome, or fatal arrhythmia	*UGT2B7* (rs7668258)
10.	Liu et al. (2018) [16]	Cancer Hospital, Chinese Academy of Medical Sciences, Beijing, China.	*N* = 147 Retrospective study	Cases − 50 (32–72), Controls − 51 (30–75)	Epirubicin 90 mg/m^2^ for 4 cycles, cumulative dose: 360mg/m^2^ Epirubicin 75 mg/m^2^ for 6 cycles, cumulative dose: 450 mg/m^2^	MALDI-TOF mass spectrometry	ST-T segment abnormalities, elevated myocardial enzymes, arrhythmia, and QRS pat tern or duration abnormalities	*ATM* (rs1003623, rs227060, rs228589, rs664143, rs664677), *ATG 5* (rs473543 and rs3761796), *ATG 7* (rs2594971, rs111595248 and rs4684789), *ATG12* (rs1058600 and rs5870670), *ATG13* (rs13448, rs10838611), *MAP1LC3A* (rs4911429 and rs6088521), *MAP1LC-3B* (rs9903, rs35227715, rs7865, and rs16944733), *CASP3* (rs1049216, rs12108497, rs2720376), *CRYAB* (rs14133), and *STMN1* (rs182455).
11.	Ruiz-Pinto et al. (2018). [29]	La Paz University Hospital, Madrid, Spain	*N* = 61 (cases = 18, Controls = 43)	Cases: median 59.5 (36–72), Controls: median 49 (27–73)	Doxorubicin cumulative dose in cases: 298.4 (200–588) mg/m^2^ Doxorubicin cumulative dose in controls: 298.6 (150–375) mg/m^2^	Illumina array	Cardiac failure grade 3–5, Asymptomatic decrease of left ventricular ejection fraction (LVEF) ≥10%.	*ETFB* rs79338777
12.	Nyangwara V et al. (2022) [33]	University of Zimbabwe, Zimbabwe.	*N* = 50, Prospective study	48.0 (44.5–59.0)	Doxorubicin cumulative dose 238.89 mg/m^2^	TaqMan genotyping,	LVEF threshold of greater than 10% reduction from the normal echocardiograms (normal ≥ 60%)	*SLC28A3* (rs7853758), *UGT1A6* (rs17863783) *RARG* (rs2229774)
13.	Norton et al. (2020). [19]	N9831 trial- Multicenter study (United States): Mayo Clinic Biobank sites in Jacksonville, Florida, and Rochester, Minnesota.	*N* = 1010, Retrospective nested case–control study	76.0 (61–81)	Doxorubicin 60 mg/m² every 3 weeks for 4 cycles cumulative dose:240 mg/m²	Sanger sequencing	Symptomatic CHF, definite cardiac death because of myocardial infarction, CHF or arrhythmia, or probable cardiac death without documented etiology	*TRPC6* (rs77679196),
14.	Todorova et al. (2017). [36]	University of Arkansas for Medical Sciences, USA.	*N* = 30, case–control genetic study.	53.1 (35–76)	Doxorubicin cumulative dose: 240 mg/m^2^	Illumina array	A decline of LVEF by > 10% or below 55% was considered abnormal.	*NFKBIL1*,* TNF-α*,* ATP6V1G2*,* MSH5*,* MICA*,* LTA*,* BAT1*,* NOTCH4,HLA*
15.	Vaitiekus et al. (2021) [18]	Lithuanian University of Health Sciences (LUHSH), Kaunas, Europe	*N* = 81, Prospective study	Case: 52.9 ± 10.29, Controls: 54.8 ± 9.02	Doxorubicin cumulative dose in Cases : 241 (146–248) mg/m^2^ Doxorubicin cumulative dose in controls :239(150–300) mg/m^2^.	TaqMan genotyping	Reduction in LVEF is ≥ 10% from baseline to ≤ 55%, without accompanying signs or symptoms defined as Subclinical Cardiotoxicity.	*HFE* (rs1799945), *HFE* (rs1800562)
16.	Vivenza et al. (2013). [35]	Not Reported	*N* = 48, Prospective study	57.5 (28–73)	Epirubicin cumulative dose: 3.60 mg/m^2^	TaqMan genotyping	Development of overt CHF (grade III) or a decline of LVEF below 50% (grade II) at any time point during the 3-year follow-up	*AGT* (rs4762), *AGT* (rs699), *ACE* (rs4340), *CYP11B2* (rs1799998), *AGTR1*(rs5186), *GSTM1*, *GSTT1*, *GSTP1* (rs1695), *TP53* (rs1042522)
17.	Volkan-Salanci et al. (2012). [34]	Not Reported	*N* = 70 (Breast cancer = 54, Lymphoma = 10), Prospective Study	49.1 ± 13.6	Doxorubicin dose cumulative dose: 317.1 ± 94.9 mg/m^2^	TaqMan genotyping	LVEF decrease > 10%; LVEF ≤ 50%	*CBR3 (rs1056892)*,* GSTP1*
18.	Vulsteke et al. (2015). [28]	Leuven Multidisciplinary Breast Cancer Center, University Hospitals Leuven, Belgium.	*N* = 877, Retrospective cohort study	50.3 ± 9.5	Epirubicin 100 mg/m^2^ 3–6 Cycles cumulative dose: 300–600 mg/m^2^	MALDI-TOF mass spectrometry	Asymptomatic decrease of Left Ventricular ejection fraction (LVEF) > 10% and cardiac failure grade 3–5 (CTCAE 4.0)	*ABCC1* (rs3743527, rs4148350, rs4551140, rs246221), *ABCC2* (rs280440, rs8187710), *CYBA* (rs4673), *NCF4* (rs4673), *RAC2* (rs13058338), *SLC28A3* (rs7853758)

**Table 3:** Summary of the significant genetic variant associations and AIC risk with Odds Ratios (OR) and 95% Confidence Intervals (CI) reported

S.no	Gene	SNP (rsID)	Genetic Model	Genotype Comparison	OR (95% CI)	*p*-value	Reference allele	References
1.	*ABCC1*	rs4148350	Dominant model	TG vs. GG	9.661 (1.418–65.82)	0.0021	G	Gintare Muckiene et al. (2023) [13].
		rs246221	Heterozygous comparison	TC vs. TT	1.59 (1.07–2.35)	0.021	T	Vulsteke et al. (2015) [28].
2.	*ATP6V1G*	rs2071594	Allelic model	C/G	6.83	0.02	G	Todorova et al. (2017) [36].
		rs3130059	Allelic model	G/C	6.83	0.02	C	
		rs11796	Allelic model	T/A	4.12	0.03	A	
3.	*BAT1*	rs2239527	Allelic model	G/C	4.13	0.05	C	Todorova et al. (2017) [36].
4.	*C6orf10*	rs2050190	Allelic model	G/A	3.89	0.02	A	Todorova et al. (2017) [36].
5.	*CBR3*	rs1056892	Additive model	GG = 0, GA = 1, AA = 2	2.50 (1.22–5.11)	0.012	G	Hertz et al. 2016 [24]
6.	*ETFB*	rs79338777	-	-	14.1 (1.60–124)	0.0079	C	Ruiz-Pinto et al. (2018) [29].
7.	*GPX1*	rs1050450	Recessive model	CC vs. (CT + TT)	2.345	0.007	C	Kopeva et al. (2022) [20].
8.	*HLA-C*	rs9264942	Allelic model	G/A	8.61	0.01	A	Todorova et al. (2017) [36].
		rs2523619		G/A	6.56	0.01	A	
		rs10484554		A/G	5.41	0.04	G	
9.	*HFE (H63D)*	rs1799945	Dominant model	GG + GC vs. CC	3.57 (1.06–8.59)	0.014	C	Vaitiekus et al. (2021) [18]
10.	*LTA*	rs909253	Allelic model	G/A	6.83	0.02	A	Todorova et al. (2017) [36].
		rs1041981		A/C	6.83	0.02	C	
11.	*MICA*	rs2523451	Allelic model	A/G	4.50	0.04	G	Todorova et al. (2017) [36].
12.	*MSH5*	rs3131379	Allelic model	A/G	11.58	0.04	G A	Todorova et al. (2017) [36].
		rs3131378		G/A				
13.	*NADPH oxidase*	rs4673	Homozygote comparison	TT	2.7529 (1.30–5.80)	0.0077	C	Grakova et al. (2021) [31].
		rs4673	Homozygote comparison	TT	2.753	0.008	C	Kopeva et al. (2022) [20].
14.	*NFKBIL1*	rs2071591	Allelic model	A/G	6.83	0.02	G	Todorova et al. (2017) [36].
		rs3093949		A/G	8.87	0.01	G	
		rs2071592		A/T	7.99	0.01	T	
15.	*NOTCH4*	rs3134931	Allelic model	G/A	0.22	0.05	A	Todorova et al. (2017) [36].
16.	*NOS3*	rs1799983	Homozygote comparison	TT	3.0585 (1.20–7.73)	0.0182	G	Grakova et al. (2021) [31].
17.	*TNF- α*	rs1800629	Allelic model	A/G	5.67	0.03	G	Todorova et al. (2017) [36].
18.	*TRPC6*	rs77679196	Allelic Model	Frequency of “A” allele	12.84	0.032	A	Advani et al. (2023) [17].
					2.01	0.002	A	Norton et al. (2020) [19].

**Table 4:** Protective genetic variants associated with reduced risk of Anthracycline-Induced Cardiotoxicity (AIC)

S.no	Gene	SNP (rsID)	Genetic Model	Genotype Comparison	OR (95% CI)	*p*-value	Reference allele	References
1.	*ABCB1*	rs1045642	Additive model	CC = 0, CT = 1, TT = 2	0.48 (0.23–1.00)	0.049	C	Hertz et al. (2016) [24].
2.	*NCF4*	rs1883112	Dominant model	AA + AG vs. GG	0.486 (0.272–0.867)	0.015	A	Domas Vaitiekus et al. (2025) [14].
3.	*UGT2B7*	rs7668258	Additive model	CC-0, CT-1, TT-2	0.259 (0.103–0.651)	0.0004	C	Li et al. (2019) [30]

### Study characteristics

A total of 18 candidate gene association studies were found to be eligible for qualitative synthesis, involving 4703 breast cancer patients (Fig. 1). Race/Ethnicity of the study participants has been reported in four studies [15, 17, 24, 36]. Of the identified 18 studies, two studies were conducted in the Asian continent, both of which were from China [16, 30]. Five studies were conducted in North America, of which four were conducted in the United States [17–19, 24] and one study was conducted in New York [15] Five studies were conducted in Europe, of which three were from Lithuania [13, 14, 18] ,one from Belgium [28], and one from Spain [29]. Two studies were conducted in the African region, one in Zimbabwe [33] and one in Egypt [32]. Two studies were conducted in Russia [20, 31]. While the other two studies did not report the location of the study [34, 35].

### Anthracycline dose

Doxorubicin (*n* = 14) and Epirubicin (*n* = 4) are the common anthracyclines reported in the included studies. The cumulative doxorubicin doses range from approximately 120 mg/m² to 366 mg/m². Median values for cases were reported between 239.5 mg/m² and 238 mg/m², while median values for controls ranged from 236 mg/m² to 239 mg/m². Several studies reported a fixed regimen of 240 mg/m² (60 mg/m² every 3 weeks for 4 cycles) [15, 17, 19, 24, 36], whereas others reported higher cumulative exposures, with mean values up to 317 ± 94.9 mg/m² [34], and a median of 360 mg/m² [31]. The reported cumulative Epirubicin dose ranges from 300 to 600 mg/m² for 3–6 cycles, with a median of 302.0 mg/m² (IQR: 281.0–321.0) across the regimens.

### Outcome measures

Across the 18 included studies, cardiotoxicity has been defined with both objective measures and subjective clinical outcomes. Objective criteria most commonly involved echocardiography or MUGA-based left ventricular ejection fraction (LVEF) decline, with a threshold such as an absolute decline of ≥ 10% [14, 18, 20, 28–31, 33, 34, 36] from baseline to values below 55% [14, 15, 24, 36] or ≥ 5% with concurrent symptoms or ≥ 10% without symptoms as per Cardiac Review and Evaluation Committee (CREC) criteria [15]. The lower cut-offs of LVEF vary across studies, like the decline to < 53% [13, 30] or ≤ 50% [34]. Additional objective markers included ST–T sgment changes, QRS abnormalities, arrhythmia on ECG, and elevated myocardial enzymes [16].

Cardiotoxicity was defined based on subjective outcomes in some studies, such as symptomatic congestive heart failure (CHF), acute coronary syndrome, fatal arrhythmia, and probable or definite cardiac death [17, 19, 30] Several studies distinguished subclinical cardiotoxicity from overt events [35], while others incorporated grading criteria from the National Cancer Institute Common Toxicity Criteria (NCI CTC)(version 2.0) [35], Common Terminology Criteria for Adverse Events (CTCAE) [28, 29, 32] American Society of Echocardiographers Guidelines [24].

### Genotyping technique

Various genotyping techniques have been reported across the studies, such as the TaqMan genotyping assay (*n* = 8) [13–15, 18, 32–35], MALDI-TOF mass spectrometry (*n* = 4) [16, 17, 24, 28], pyrosequencing (*n* = 1) [30], Sanger sequencing (*n* = 1) [19], Polymerase Chain reaction (*n* = 2) [20, 31] and Illumina genotyping arrays (*n* = 1) [29, 36].

### Results of the individual studies

**Fig. 2:**
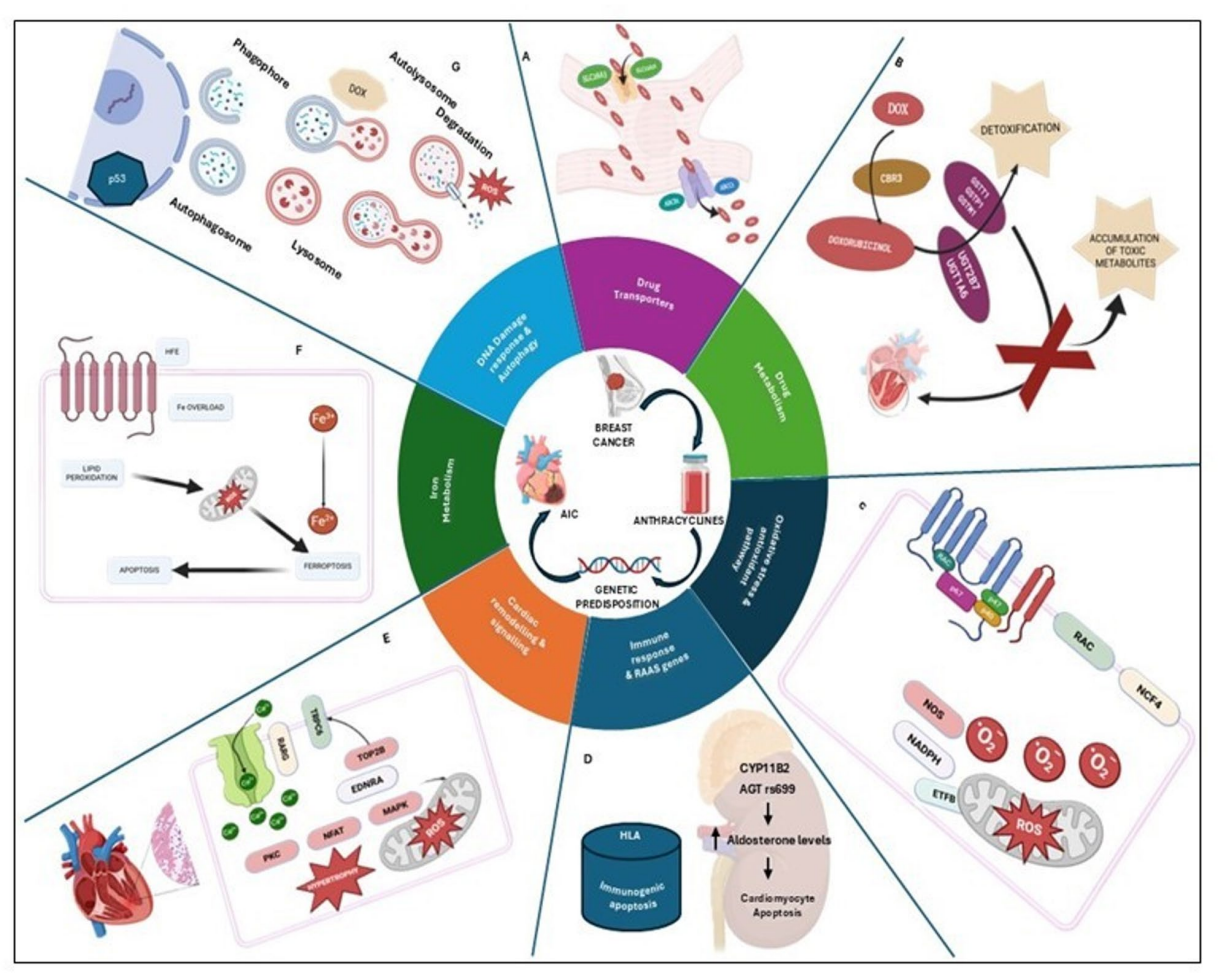
Candidate gene determinants involved in biological pathways of AIC. **A**. *CBR3*, *GSTP1*, *GSTM1*, and *GSTT1* modulate the enzymatic reduction and detoxification of anthracyclines. **B**. Genes such as *ABCB1*, *ABCC1*, *SLC28A3*, and *SLC22A16* regulate the intracellular accumulation of anthracyclines. Variations in these genes can alter drug efflux and uptake. **C**. Genes such as *NOS*, *NADPH RAC*, *NCF4*, *ROS* regulators, and *ETFB* are involved in the generation and detoxification of reactive oxygen species. **D**. *HFE* gene variants influence iron homeostasis, promoting iron-mediated free radical generation. **E**. Genes such as *TOP2B* and *p53* mediate anthracycline-induced DNA strand breaks and apoptotic signalling, contributing tocardiomyocyte death. **F**. *RARG*, *TRPC6*, *EDNRA*, *NFAT*, and *PKS* regulate hypertrophic signalling that remodels cardiac tissue following injury. **G**. Variants in *HLA*, *AT1R*, *ANG II*, *CYP11B2*, and *AGT* rs699 modulate inflammatory and *RAAS*, influencing immune-mediated injury and cardiac remodelling

The genetic variations involved in various biological pathways influence AIC in various ways. The detailed mechanism is illustrated in Fig. 2. The findings from individual studies were synthesized and grouped based on the mechanism.

#### Drug transporters

##### ATP-binding cassette transporters

Muckienė et al. reported that the *ABCC1* rs4148350 variant leads to AIC in breast cancer patients, with TG carriers showing a higher risk compared to GG carriers (OR 9.661, 95% CI 1.418–65.824, *p* = 0.0021) [13]. In contrast, Vulsteke et al. did not observe a statistically significant association for this variant [28]. However, in the same study, it was found that the heterozygous T-allele carriers of the *ABCC1* rs246221 were prone to be at a higher risk of LVEF decline by about 10% compared to homozygous TT carriers (OR 1.59, 95% CI 1.1–2.3, *p* = 0.02) [28].

Hertz et al. found that the *ABCB1* rs1045642 protects against cardiac injury (OR 0.48, 95% CI 0.23–1.00, *p* = 0.049) [24]. However, this variant did not reach statistical significance in the study by Muckiene et al. [13]. Other ABC transporters, such as *ABCC1* rs4551101 [28], *ABCC1* rs3743527 [24] did not reach statistical significance.

##### Solute carriers

*SLC28A3* rs7853758 did not demonstrate a statistically significant correlation with AIC in three studies [24, 28, 33]. An odds ratio with a broad confidence interval but a consistent trend (additive model: OR 0.55, 95% CI 0.16–1.9, *p* = 0.43) was reported by Hertz et al. [24]. Similarly, *SLC22A16* rs714368, rs6907567, rs723685, rs12210538 variants are not associated with AIC [24].

#### Drug metabolizing enzymes

##### Carbonyl Reductases (CBR)

Hertz et al. reported that the *CBR3* rs1056892 variant increases the risk of decline in LVEF to less than 55% (additive model: OR 2.50, 95% CI 1.22–5.11, *p* = 0.012), with greater risk after considering covariates (*p* = 0.008–0.048) [24]. Lang et al. found that doxorubicin treatment disrupts cardiac systolic function across all CBR3 genotype groups (F [1,89] = 50.33, *p* < 0.001). with greater LVEF decline in GG carriers, moderate LVEF decline in AG carriers, and a non-significant LVEF decline in AA carriers (*p* = 0.072) [15]. A similar trend of genotype-dependent decline in LVEF has been reported by Volkan-Salanci et al. (GG vs. AA: *p* = 0.039) [34]. However, the association did not reach statistical significance in the study conducted by Domas Vaitiekus et al. (AA vs. GG, OR 0.597, 95% CI 0.068–5.212, *p* = 0.640) [14]. Similarly, in NSABP B-31, the *CBR3* rs1056892 variant was more frequently present in cases than in controls, but did not reach statistical significance (0.75 vs. 0.62; OR 1.82, 95% CI 0.65–5.26, *p* = 0.259) [17].

In the Egyptian breast cancer cohort, *CBR1* rs20572 was associated with significantly higher exposure levels of doxorubicin [32]. While *CBR1* rs9024 revealed no significant association with AIC (AG vs. GG: OR 1.49, 95% CI 0.30–7.40, *p* = 0.624) in a study by Vaitiekus et al. [14].

##### Uridine 5′-diphospho-GlucuronosylTransferases (UGT)

Li et al. reported the function of *UGT2B7-161* rs7668258 in breast cancer patients undergoing epirubicin chemotherapy. Multivariate logistic regression showed that the − 161 T allele was independently associated with lower risk of cardiotoxicity (additive model: CC-0, CT-1, TT-2, OR 0.259, 95% CI 0.103–0.651, *p* = 0.0004) [30]. Trastuzumab administration, higher cumulative doses of epirubicin, and elevated cardiac biomarker concentrations were identified as independent risk factors for cardiotoxicity [30]. *UGT1A6* rs17863783 did not reach statistical significance in three studies [14, 24, 33].

##### Glutathione S-Transferases (GST)

Volkan-Salanci et al. reported no initial variation in cardiac parameters across *GSTP1* genotypes in the initial follow-up. However, at a 1-year follow-up, *GSTP1* G-allele carriers (AG + GG) had a higher risk of AIC compared to AA carriers, with lower fractional shortening (−11.3 ± 12.3% vs. −1.7 ± 10.7%; *P* = 0.024) [34], higher end systolic diameter (7.3 ± 12.1 vs. −0.9 ± 8.7%; *p* = 0.018) [34], and more frequent peak filling rate decline (85% vs. 15.4% of AA; *p* = 0.007) [34]. Vivenza et al. reported no significant correlation of *GSTT1* or *GSTP1*. But, elevated cardiotoxicity risk (*p* = 0.147) has been identified in the *GSTM1* null genotype, which did not reach conventional levels of statistical significance [35].

#### Oxidative stress & antioxidant pathways

##### Neutrophil Cytosolic Factor 4 (NCF4)

A study by Vaitiekus et al. reported a risk reduction tendency for *NCF4* rs1883112 (Dominant model: OR = 0.49, 95% CI 0.27–0.87, *p* = 0.015) [14]. In contrast, Hertz et al. and Vulsteke et al. reported no association of *NCF4* rs1883112 with AIC [24, 28].

##### Nitric Oxide Synthase 3 (NOS3)

The *NOS3* rs1799983 TT carriers are associated with higher risk of AIC (OR = 3.06, 95% CI: 1.21–7.73, *p* = 0.018) [20, 31].

##### Nicotinamide Adenine Dinucleotide Phosphate (NADPH)

The TT genotype of the *NADPH* oxidase rs4673 is associated with AIC (OR = 2.75, 95% CI: 1.31–5.80, *p* = 0.0077) [20, 31].

##### Ras-related C3 botulinum toxin substrate (RAC)

Hertz et al. reported the effect of *RAC2* rs13058338 on systolic dysfunction, which did not reach statistical significance in the additive model (OR 0.75, 95% CI: 0.31–1.82, *p* = 0.38) [24]. Similarly, Vulsteke et al. did not observe any significant association for this variant [28].

##### Glutathione Peroxidase 1 (GPX1)

Kopeva et al. identified that the *GPX1* rs1050450 CC genotype was associated with AIC (OR = 2.345, *p* = 0.007) and suggested it as a potential genetic marker for risk assessment before chemotherapy [20].

##### Electron transfer Flavoprotein subunit Beta (ETFB)

The *ETFB* rs79338777 minor T allele carriers are at risk of chronic AIC in breast cancer cohort (discovery cohort) which later replicated in childhood cancer cohort (replication cohort) when analysed separately (discovery cohort: OR 14.1, 95% CI 1.60–124, *p* = 0.017; replication: OR 6.17, 95% CI 1.61–27.7, *p* = 7.97 × 9 × 10^− 3^) [29].

The other explored variants of Oxidative Stress & Antioxidant Pathways, such as *CYBA* rs4673 [24, 28], *CYBA* rs1049255 [14] and *SOD2* rs4880 [20], *PON1* rs662 (AG vs. AA, OR 0.724, 95% CI: 0.246–4.946, *p* = 0.80) [14] did not reach statistical significance in breast cancer patients.

#### Cardiac remodelling & signalling

##### Transient receptor potential cation channel subfamily C (TRPC6)

Advani et al. identified a significant association of *TRPC6* rs77679196 with anthracycline-induced cardiotoxicity (AIC) in the NSABP B-31 trial (OR = 12.84, 95% CI 1.24–133.2; *p* = 0.032). The massive effect estimate was accompanied by wide confidence intervals. This can be attributed to the small number of cardiac events in the analysed NSABP B-31 chemotherapy sub-cohort (*N* = 10). In an independent cohort from the Mayo Clinic Biobank, Norton et al. reported that the *TRPC6* rs36111323 variant was significantly more frequent among heart failure cases than among anthracycline/trastuzumab-exposed controls (OR = 2.01; *p* = 0.002), after adjustment for sex and hypertension.

*RARG* rs2229774 is not associated with AIC risk (*P* = 0.471) in a study by Nyangwara V et al. [33]. Similarly, Hertz et al. demonstrated a non-significant association of *TOP2B* rs10865801 with AIC (Additive model: OR 1.32, 95% CI 0.67–2.61, *P* = 0.47) [24]. Advani et al. evaluated variants in *BRINP1* rs62568637, *LDB2* rs55756123 (OR 1.93, 95% CI 0.25–14.7, *p* value = 0.525), and *RAB22A* rs707557 (OR 1.28, 95% CI 0.51–3.12, *p* value = 0.598), but none of these demonstrated significant associations [17]. Investigations of *EDNRA* rs5335 did not reach statistical significance [20, 31].

#### Iron metabolism genes

##### Homeostatic iron regulator (HFE)

The G allele carriers of *HFE* rs1799945 have an increased risk of developing AIC (OR = 3.44, 95% CI 1.40–8.47, *p* = 0.005). In contrast, the *HFE* rs1800562 variant is not statistically significant [18].

#### DNA damage response and cell cycle regulation

Kopeva et al. identified that AIC was significantly associated with the *TP53* arginine genotype (OR = 2.97, *p* = 0.001), while the proline genotype appeared to have a protective effect (OR = 0.126, *p* = 0.028) [20]. Similarly, Vivenza et al. reported that the patients carrying the GC (p.Pro72Arg, 42%) and GG (p.Arg72Arg, 50%) genotype showed a higher prevalence of hypertension compared to those with the CC (p.Pro72Pro, 8%) genotype (*p* = 0.180). The association was borderline significant when GC and GG carriers were grouped and compared with CC patients (*p* = 0.077) [35].

In a study by Liu et al., the frequency of cardiac events did not reach statistical significance in *ATM* variants such as *ATM* rs1003623 (χ² = 1.57, *p* = 0.46) *ATM* rs227060 (χ² = 0.85, *p* = 0.65) *ATM* rs228589 (χ² = 0.82, *p* = 0.57) *ATM* rs664143 (χ² = 0.20, *p* = 0.90) and *ATM* rs664677 (χ² = 0.95, *p* = 0.62) [16].

Variants in *CASP3*, such as *CASP3* rs1049216 (χ² = 1.75, *p* = 0.42), *CASP3* rs12108497 (χ² = 0.97, *p* = 0.61), *CASP3* rs2720376 (χ² = 0.46, *p* = 0.79), in breast cancer patients receiving anthracyclines did not reach significance [16].

Similarly, *CRYAB* rs14133 (χ² = 6.31, *p* = 0.10), and *STMN1* rs182455 (χ² = 2.84, *p* = 0.24) did not demonstrated any statistical significance. Kopeva et al. reported insignificant association of AIC with *CASP8* variants such as *CASP8* rs3834129 (χ² = 0.002, *p* = 0.961) *CASP8* rs1045485 (χ² = 0.073, *p* = 0.786) [20].

#### Autophagy & apoptosis genes

In a study by Liu et al., Chi-square analysis did not reveal any association between *ATG* gene variants and AIC. In *ATG5*, neither *ATG5* rs473543 (χ² = 1.42, *p* = 0.49) nor *ATG5* rs3761796 (χ² = 2.06, *p* = 0.36) indicated any significant association. Similarly, *ATG7* rs2594971 (χ² = 0.37, *p* = 0.83), *ATG7* rs111595248 (χ² = 0.33, *p* = 0.56), and *ATG7* rs4684789 (χ² = 0.50, *p* = 0.78), were not correlated with cardiotoxicity [16].

Liu et al. observed that carriers of the G allele at *ATG13* rs10838611 have abnormal electrocardiogram findings (OR = 2.26, 95% CI = 1.32–3.87; *p* < 0.01), indicating a higher probability of cardiac dysfunction. *MAP1LC3A* rs4911429 (χ² = 1.17, *p* = 0.56), *MAP1LC3A* rs6088521 (χ² = 0.67, *p* = 0.72) and *MAP1LC3B* rs9903 (χ² = 0.55, *p* = 0.76), *MAP1LC3B* rs35227715 (χ² = 0.53, *p* = 0.77), *MAP1LC3B* rs7865 (χ² = 0.57, *p* = 0.45), or *MAP1LC3B* rs16944733 (χ² = 1.58, *p* = 0.45) are not significantly associated with cardiac events [16].

#### Inflammatory and immune response genes

Todorova et al. carried out a candidate gene association study in breast cancer patients treated with doxorubicin and identified nine genes with eighteen genetic variants, including the human leucocyte antigen region, associated with abnormal decline in LVEF. The authors reported the estimated odds ratio for the development of cardiotoxicity for the minor allele, with the major allele as reference, although confidence intervals were not provided significant associations were reported for variants in *HLA-C* rs9264942 (OR = 8.61, *p* = 0.01), *HLA- C* rs2523619 (OR = 6.56, *p* = 0.01), *HLA-C* rs10484554 (OR = 5.41, *p* = 0.04) [36].

*NFKBIL1* variants such as *NFKBIL1* rs2071591 (OR = 6.83, *p* = 0.02), *NFKBIL1* rs3093949 (OR = 8.87, *p* = 0.01), *NFKBIL1* rs2071592 (OR = 7.99, *p* = 0.01), which have been associated with inflammatory and autoimmune disorders like Myocardial infarction [37] Graves’ disease [38], and rheumatoid arthritis [39] were also associated with AIC [36].

Additionally, AIC associations were reported for *C6orf10* rs2050190 (OR = 3.89, *p* = 0.02), *TNF-α* rs1800629 (OR = 5.67, *p* = 0.03), *MSH5* rs3131379 (OR = 11.58, *p* = 0.04), *MSH5* rs3131378 (OR = 11.58, *p* = 0.04), *MICA* rs2523451 (OR = 4.50, *p* = 0.04), *LTA* rs909253 (OR = 6.83, *p* = 0.02) [36], *LTA* rs1041981 (OR = 6.83, *p* = 0.02), *BAT1* rs2239527 (OR = 4.13, *p* = 0.05), *NOTCH4* rs3134931 (OR = 0.22, *p* = 0.05), *ATP6V1G* rs2071594 (OR = 6.83, *p* = 0.02), *ATP6V1G* rs3130059 (OR = 6.83, *p* = 0.02) and *ATP6V1G* rs11796 (OR = 4.12, *p* = 0.03) [36].

#### Renin–Angiotensin–Aldosterone system (RAAS) genes

In a study by Vivenza et al., the baseline serum aldosterone levels showed a significant difference among *CYP11B2* rs1799998 genotypes (*p* = 0.03), with the highest levels in CT, intermediate in TT, and lowest in CC carriers [35] The small cohort size limited the assessment of other cardiotoxicity outcomes in this study [35].

The *ACE* rs4340 variant was associated with higher diastolic blood pressure (DBP) and serum aldosterone levels (*p* = 0.04 and *p* = 0.003, respectively). Similarly, the *AGT* rs699 polymorphism is also correlated with serum aldosterone, with higher levels in CC carriers than CT or TT carriers (*p* = 0.045) [35].

No associations were observed for *AGTR1* A1166C or *AGT* p.Thr174Met with aldosterone or blood pressure [35].

## Discussion

The current systematic review explored the genetic association of breast cancer patients undergoing anthracycline chemotherapy. As the overall survival rate for patients with breast cancer has increased, it is crucial to preserve and improve their quality of life. One of the major side effects of treatment that can still have an impact on long-term results is cardiotoxicity.

Pharmacogenomic variants modulate anthracycline-induced cardiotoxicity (AIC) in breast cancer patients through gene-specific gain or loss-of-function effects that alter drug transport, drug metabolism, redox homeostasis, calcium signalling, and DNA damage response. For example, the *ABC* transporter gene, *ABCC1*, was first discovered in a doxorubicin-resistant small cell lung cancer cell line that did not overexpress P-glycoprotein [40]. This family predominantly exerts a gain-of-function effect, known to influence the pharmacokinetics of anthracyclines by enhancing efflux activity, which leads to drug resistance in cancer cells [41]. Similarly, Anthracycline metabolism plays a key role in AIC. The CBR3 rs1056892 variant makes doxorubicin more susceptible to anthracycline-induced cardiotoxicity by increasing its catalytic conversion to the cardiotoxic metabolite doxorubicinol [10]. Similarly, *UGT2B7*, a key enzyme responsible for the glucuronidation and elimination of anthracycline metabolites, exhibits a protective effect where the rs7668258 variant is associated with a reduced cardiotoxic risk due to improved detoxification efficiency [42].

The other widely studied mechanism of AIC includes the production of oxygen-free radicals that damage the heart. While *RAC2*, *NADPH* oxidases (NOX), uncoupled nitric oxide synthase, and mitochondria are major enzymatic sources for reactive oxygen species generation [43] the *NCF4* rs1883112 polymorphism, due to its loss-of-function effect, inhibits oxidase activation and reduces the formation of reactive oxidant intermediates [14] and exhibits cardioprotective effect.

The iron metabolism genes, such as the *HFE* rs1799945 variant, that are involved in the intracellular metabolism of doxorubicin, are also associated with the generation of reactive oxygen intermediates through a gain-of-function effect [18]. Similarly, the increase in calcium ion influx due to the *TRPC6* mutation leads to cardiac hypertrophy [17]. Genes such as *ATM* kinase play a crucial role in response to DNA damage and are associated with the response to oxidative stress [44]. Furthermore, the pharmacogenetic contributions of genes involved in inflammatory and immune responses, the RAAS system, and autophagy and apoptosis pathways require further exploration.

In this current review, breast cancer patients with *ABCC1* variants, such as the rs4148350 TG genotype [13] and the rs246221 heterozygous T-allele carriers [28], were at increased risk of AIC. Similarly, the GG carriers of the *CBR3* rs1056892 variant had a higher risk of decline in LVEF following anthracycline therapy [15, 24, 34]. In a one-year follow-up, G-allele carriers of the *GSTP1* polymorphism have lower fractional shortening, higher end-systolic diameter, and a decline in peak filling rate [34].

Similarly, it has been found that the TT carriers of *NOS3* rs1799983 [20, 31], *NADPH* rs4673 [20, 31], are prone to cardiac dysfunction, while the *GPX1* rs1050450 CC genotype represents a loss-of-function antioxidant variant increasing susceptibility to oxidative injury [20].The minor T allele of *ETFB* rs7938777 can also serve as a marker for cardiotoxic risk assessment [29]. The higher frequency of the *TRPC6* variant has been observed in heart failure cases [19].

In iron metabolism genes, the G allele of *HFE* rs1799945 has been associated with AIC [14]. In the class of DNA damage response genes, carriers of the *TP53* gene with the GG or GC nucleotide sequence are at risk of developing hypertension [35]. The G allele of the *ATG13* gene, related to autophagy and apoptosis, has been linked to echocardiographic changes [16]. The CT carriers of *CYP11B2* rs1799998 are associated with an increase in serum aldosterone levels, and the variant of *ACE* rs4340, AGT rs699, raises diastolic blood pressure [35]. Across nine genes, eighteen genetic variants in the *HLA* region have been associated with a fall in LVEF [36].

In contrast, genetic variants of *UGT2B7*−161 rs7668258 [30], *NCF4* rs1883112 [14], and *TP53* [20] have been associated with the risk reduction of AIC.

Understanding the genetic variants in anthracycline pathways may inform future therapeutic strategies. For instance, statins, due to their anti-inflammatory and antioxidant effects, have been investigated as potential cardioprotective agents for cardiomyopathy [45]. Similarly, dexrazoxane impairs the formation of iron compounds and oxygen-free radicals [46].

In addition to breast cancer, anthracyclines are also widely used in childhood cancers and haematological malignancies. Several studies have also reported the genetic predisposition of AIC in this cohort. Previous associations of *ABCC2* rs8287710 that have been reported in haematological malignancies [47], and *ABCC1* rs3743527, linked to decreased LVEF in childhood acute lymphoblastic leukaemia [48], did not reach significance in the breast cancer cohort. Similarly, *SLC28A3* rs7853758 has been strongly associated with AIC in other cancer types (OR 0.35, *p* = 0.00008) [23] has not reached statistical significance in breast cancer patients [24, 28, 33]. For the *CBR3* rs1056892 variant the similar findings were observed across both breast cancer [24, 34] and haematological malignancy cohorts (OR 1.79, *p* = 0.02) [10].

Following doxorubicin therapy in childhood leukaemia, heterozygous *HFE* rs1800562 carriers showed recurrent increases in cardiac troponin T and decreased left ventricular function [49]. In a Spanish cohort, anthracycline exposure and the *HFE* haplotypes were also associated with iron accumulation [50]. However, rs1800562 did not significantly correlate with AIC risk in breast cancer patients [18]. The other gene, *RAC2*, encodes a GTPase essential for *NADPH* oxidase activity [51, 52]. But, the *RAC2* rs13058338 variant that is associated with congestive heart failure in haematological malignancies (OR 2.8, 95% CI: 1.4–5.6) [47], did not show statistically significant associations in breast cancer patients [24, 28]. While level B (moderate) evidence through a systematic evidence synthesis approach supports pharmacogenomic testing for *RARG* rs2229774, *SLC28A3* rs7853758, and *UGT1A6* rs17863783 in childhood cancer patients treated with doxorubicin or daunorubicin [53] did not reach statistical significance in patients with breast cancer.

This lack of replication observed in the breast cancer cohorts can be mainly attributed to population differences rather than the absence of a true genetic effect. Pharmacogenomic studies are widely influenced by minor allelic frequencies in the population. For instance, a study by Aminkeng et al. reported that the *RARG* rs2229774 variant has a minor allele frequency of approximately 25% in the European children’s cohort. It demonstrated a strong association with AIC, with an odds ratio of 4.7 (95% CI 2.7–8.3), suggesting sufficient power to detect a genetic effect [54]. However, the variant had a lower minor allele frequency of 14% in African ancestry populations, which may have limited the replication of this association in the breast cancer cohort [33].

Likewise, a protective effect is linked to the minor A allele of *SLC28A3* rs7853758. The high allele frequency of 39% in Mexican paediatric anthracycline-treated cohorts provides sufficient power to identify a protective effect against cardiotoxicity [55]. The same allele occurs at a frequency of about 16–17% in Caucasian breast cancer cohorts, as reported in BCIRG 006 [56]. This could make it comparatively difficult to replicate protective associations in this cohort. Similarly, in a study by Visscher et al., Canadian paediatric cohorts reported a strong protective association. The A allele was more frequent in controls than in cases (20% vs. 7.7%), with a combined odds ratio of 0.31 (95% CI, 0.16–0.60). However, when replicated in an independent Dutch cohort, the effect estimate did not reach statistical significance, which may be linked to the low minor allelic frequency [23].

Hence, one of the major limitations of pharmacogenomic studies is that they cannot be broadly generalizable, as the associations are strongly influenced by ancestry-specific differences in minor allele frequencies and linkage disequilibrium patterns.

The discrepancies in genetic effects across distinct populations are influenced by various factors. In the dose-stratified analysis, the protective effect of the *SLC28A3* variant is observed to be significant at higher cumulative anthracycline doses (> 250 mg/m²) (odds ratio, 0.43, *P* < 0.01), but not at lower doses (< 150 mg/m²) [57]. It implies that the genetic effect interacts with dosage exposure, and this protective association may not be statistically significant when the anthracycline dose is relatively low, which is frequently the case in many adult breast cancer protocols.

Cardiovascular comorbidities such as diabetes, hypertension, and pre-existing coronary disease are more common in adults. These risk factors can conceal subtle genetic influences that are noticeable in the paediatric population without comorbidities [58].

Additionally, anthracyclines are combined with other sequential cardiotoxic agents, such as trastuzumab, in breast cancer regimens [19], which makes it more difficult to isolate single gene-drug interactions and reduces the power to observe variant effects that are easier to detect in paediatric regimens. In retrospective analysis of breast cancer patients, the “healthy survivor” effect may decrease the apparent genetic effect, while paediatric research frequently tracks all treated patients prospectively and records a more complete incidence of cardiotoxicity.

The incidence of a clinically significant reduction in the LVEF is higher in patients receiving both trastuzumab and an anthracycline than in those receiving anthracyclines alone (36% vs. 9.5%, *p* = 0.001) [59].

Trastuzumab and radiotherapy are important modifiers of cardiotoxicity risk in breast cancer patients and can influence the interpretation of pharmacogenomic associations. Although several studies accounted for trastuzumab exposure, either through stratified analyses comparing chemotherapy alone versus chemotherapy plus trastuzumab [17, 19] or by adjusted odds ratios to trastuzumab exposure [15, 24, 34, 42]. This was not uniformly applied across all studies. In contrast, radiotherapy was considered in only a limited number of analyses [24, 28].The lack of uniform adjustment for concomitant cardiotoxic therapies likely contributes to heterogeneity and reduces the reproducibility of genetic associations in breast cancer studies.

Trastuzumab causes cardiotoxicity through a different mechanism than anthracyclines. While anthracyclines directly damage cardiac cells and generate reactive oxygen species through redox cycling and *NADPH* oxidase activity, trastuzumab interferes with HER2-mediated survival signalling in cardiomyocytes, leading to impaired protection against oxidative stress and increased mitochondrial vulnerability [60]. Because trastuzumab cardiotoxicity is primarily Type II rather than structural damage, the contribution of classic oxidative stress gene variants (such as *NOS3* or *NADPH* oxidase genes) to trastuzumab-related cardiotoxicity is less well established [61].

There is a wide heterogeneity in the definition of cardiotoxicity, ranging from an asymptomatic drop in LVEF to overt heart failure. The interpretation of genetic associations is affected by substantial variation. A drop in LVEF indicates early, subclinical myocardial damage. Symptomatic heart failure indicates a more advanced condition.

Studies that used strict clinical endpoints, such as symptomatic congestive heart failure or cardiac death [17, 19, 35] more consistently identified associations with TRPC6 and *TP53* variants. *TRPC6* plays a role in calcium signalling and cardiac hypertrophy, which supports its stronger association with overt heart failure rather than mild functional changes.

In contrast, studies defining cardiotoxicity mainly as asymptomatic LVEF decline of at least 10% [14, 18, 31] more often reported associations with genes involved in oxidative stress and drug metabolism, such as *CBR3*,* NCF4*,* HFE*, and *NOS3*. These genes are linked to early myocardial injury and subclinical cardiac remodelling, which may not immediately progress to clinical heart failure. AIC is more likely a spectrum that begins with subclinical myocardial injury and early asymptomatic declines in LVEF, progressing to clinical heart failure if untreated, which represents the notion of a pathophysiological continuum [60, 61].

**Fig. 3:**
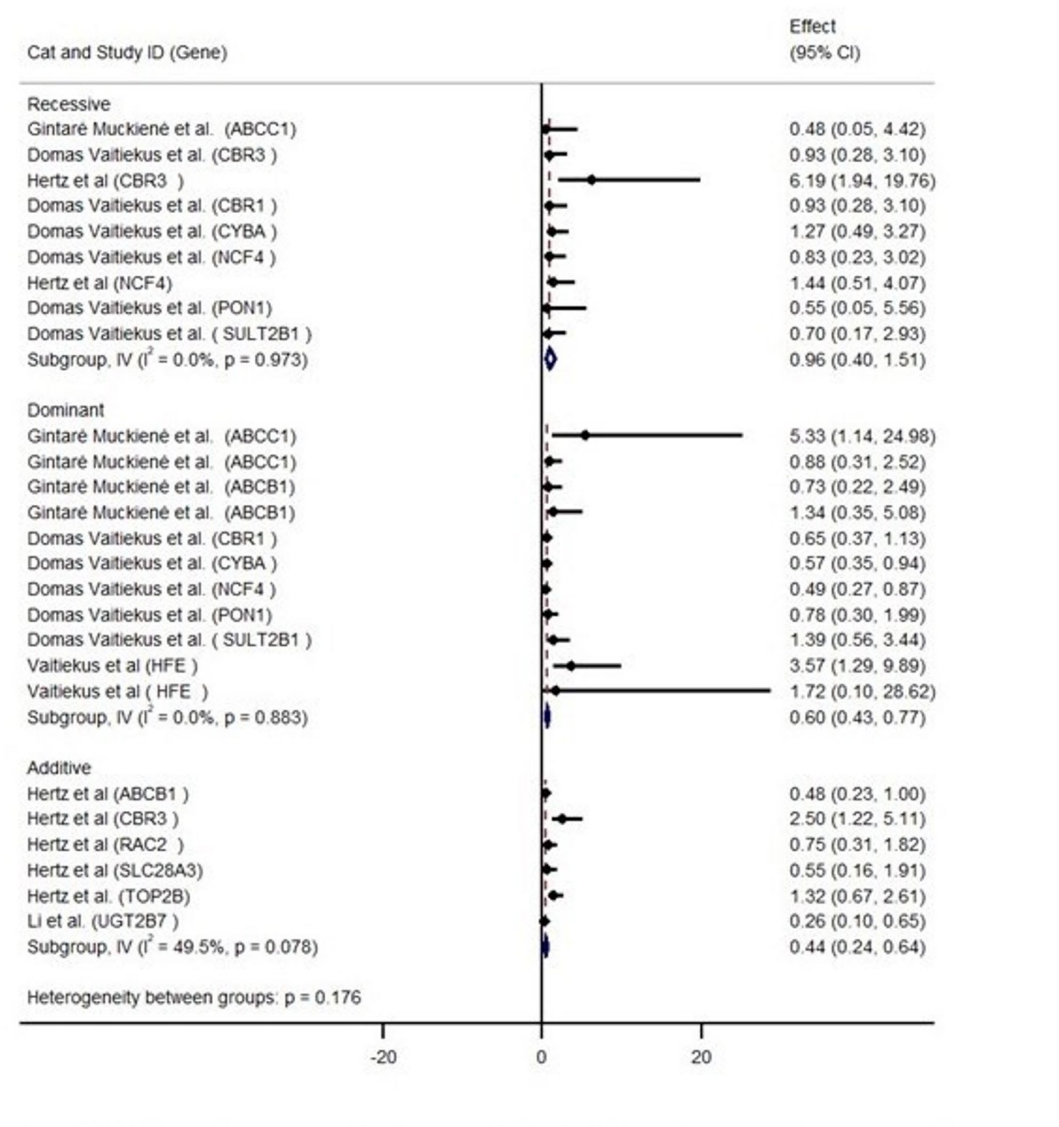
Forest plot showing odds ratios (ORs) and 95% confidence intervals (Cls) for the association between genetic variants and anthracycline-induced cardiotoxicity (AIC). ORs > 1 indicate increased risk, whereas ORs < 1 indicate a protective effect

**Fig. 4:**
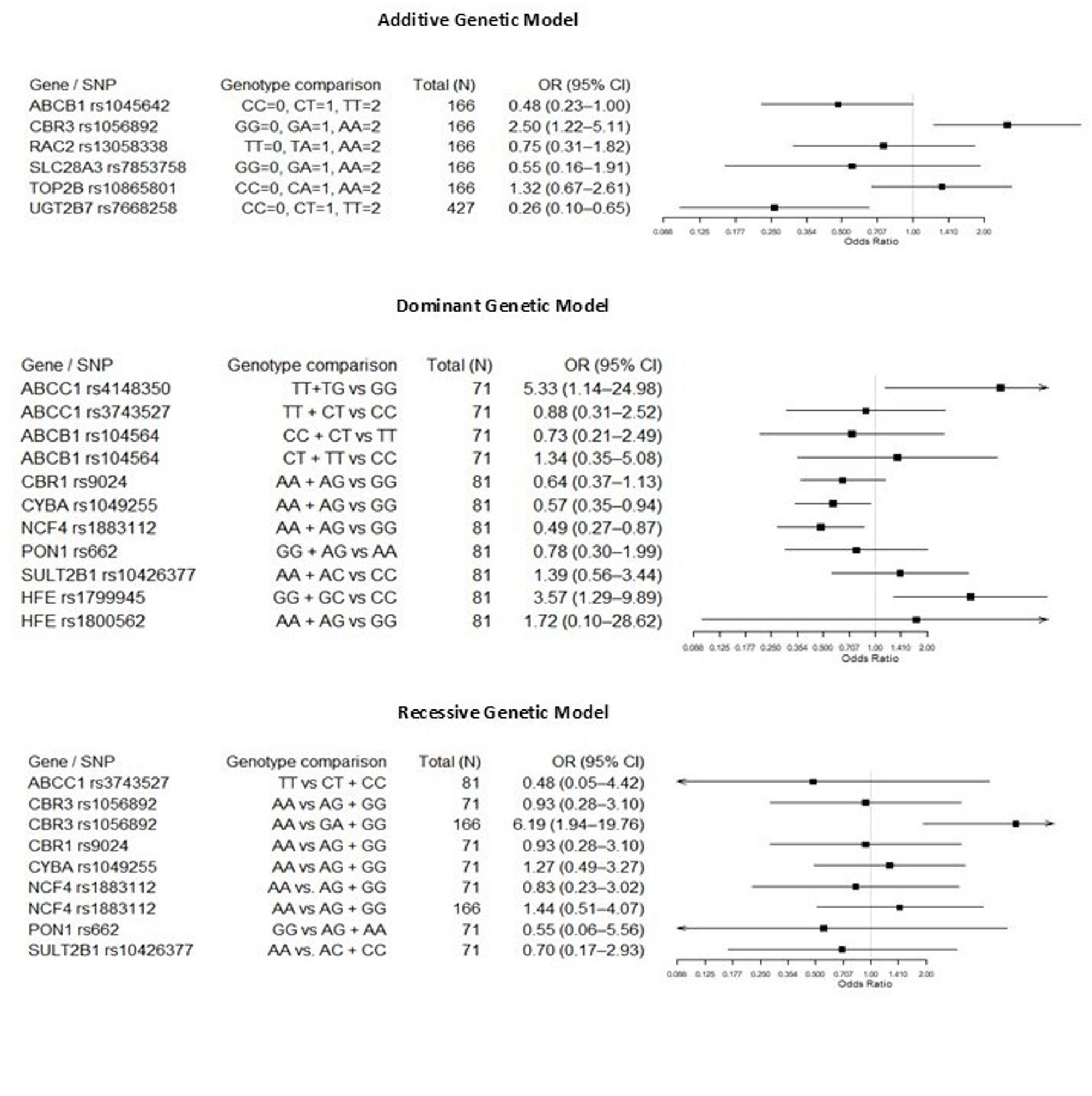
Forest plot of odds ratios (ORs) and 95% confidence intervals (cls) associated with AIC reported under Additive, Dominant, and Recessive models. ORs > 1 indicate increased risk, whereas ORs < 1 indicate a protective effect

A quantitative meta-analysis was not feasible due to substantial heterogeneity across studies. Many studies have reported genetic associations using different inheritance models and different effect estimates. These have limited the feasibility of pooling data. To address this, study-level effect sizes and heterogeneity are presented as forest plots in Figs. 3 and 4. The genetic associations investigated in *various* inheritance models are presented in *Supplementary Table 3.* We used qualitative methods to synthesize the evidence.

Despite using a rigorous and systematic approach, this study has some limitations. In pharmacogenomic research, studies with null results or small sample sizes are less likely to be published, which could lead to inflated effect estimates. Many of the included studies were underpowered and used inconsistent outcome definitions and methods to evaluate cardiotoxicity. Moreover, the genetic associations discussed represent only part of the complex biology underlying anthracycline-induced cardiotoxicity. Other relevant genetic variants, pathways, or gene-environment interactions, as well as clinical factors, may also influence AIC. Given the presence of multiple clinical risk factors for anthracycline-induced cardiotoxicity, isolating the effect of individual SNPs can be challenging. Future well-powered, multicentre prospective studies with standardized cardiotoxicity measures and comprehensive genomic analyses are needed to address these limitations.

For patients with breast cancer, a genomic perspective of AIC provides opportunities for individualised risk assessment and treatment. By identifying individuals at higher risk, clinicians can customise chemotherapy regimens or consider cardioprotective medications like dexrazoxane [4, 62]. The current anthracycline chemotherapy surveillance protocol involves baseline and serial echocardiography, and the measurement of cardiac biomarkers to identify early myocardial damage [63]. Predictive accuracy can be improved by combining the genetic risk factors with the cumulative dose of anthracycline and clinical risk factors.

## Conclusion

Genetic variations associated with various cardiotoxicity-related pathways in breast cancer patients have been summarized in the current systematic review. These variations in the drug metabolizing pathway *CBR3 rs1056892* have been consistently associated independently with AIC in four studies. Although a strong association between transporter gene variants and cardiotoxicity has been reported in breast cancer cohorts, these findings require validation in larger, ethnically diverse populations with extended follow-up. Similarly, genomic variants in different pathways explored in breast cancer patients require further investigation to reach strong evidence. While a major portion of the earlier evidence has come from childhood cancer cohorts, large-scale studies dedicated to the breast cancer population will be crucial for the validation of these findings and for translating these findings into clinical practice. While current evidence remains preliminary, the validation of genomic determinants may enable pharmacogenomic testing to guide anthracycline use in breast cancer patients.

## Supplementary Information

Supplementary Material 1.

## Data Availability

No datasets were generated or analysed during the current study.

## Abbreviations

ABCB1: ATP Binding Cassette Subfamily B Member 1
ABCC1: ATP Binding Cassette Subfamily C Member 1
ABCC2: ATP Binding Cassette Subfamily C Member 2
ACE: Angiotensin-Converting Enzyme
AGT: Angiotensinogen
AGTR1: Angiotensin II Receptor Type 1
AIC: Anthracycline-Induced Cardiotoxicity
ATG12: Autophagy Related 12
ATG13: Autophagy Related 13
ATG5: Autophagy Related 5
ATG7: Autophagy Related 7
ATM: ATM Serine/Threonine Kinase
ATP6V1G2-DDX39B: ATPase H+ Transporting V1 Subunit G2 / DEAD-Box Helicase 39B
BAT1: HLA-B Associated Transcript 1
BRINP1: BMP/Retinoic Acid Inducible Neural Specific 1
CASP3: Caspase 3
CASP8: Caspase 8
CBR1: Carbonyl Reductase 1
CBR3: Carbonyl Reductase 3
CRYAB: Crystallin Alpha B
CTRCD: Cancer Therapy-Related Cardiac Dysfunction
CYBA: Cytochrome b-245 Alpha Chain
CYP11B2: Cytochrome P450 Family 11 Subfamily B Member 2
EDNRA: Endothelin Receptor Type A
ETFB: Electron Transfer Flavoprotein Subunit Beta
GPX1: Glutathione Peroxidase 1
GSTM1: Glutathione S-Transferase Mu 1
GSTP1: Glutathione S-Transferase Pi 1
GSTT1: Glutathione S-Transferase Theta 1
HFE: Homeostatic Iron Regulator
IL1B: Interleukin 1 Beta
LDB2: LIM Domain Binding 2
LINC01060: Long Intergenic Non-Protein Coding RNA 1060
LTA: Lymphotoxin Alpha
LVEF: Left Ventricular Ejection Fraction
MAP1LC3A: Microtubule Associated Protein 1 Light Chain 3 Alpha
MAP1LC3B: Microtubule Associated Protein 1 Light Chain 3 Beta
MICA: MHC Class I Polypeptide-Related Sequence A
MSH5: MutS Homolog 5
NADPH: Nicotinamide Adenine Dinucleotide Phosphate
NCF4: Neutrophil Cytosolic Factor 4
NFKBIL1: NFKB Inhibitor Like 1
NOS3: Nitric Oxide Synthase 3
NOTCH4: Notch Receptor 4
NR1I2: Nuclear Receptor Subfamily 1 Group I Member 2
RAB22A: RAB22A, Member RAS Oncogene Family
RAC2: Rac Family Small GTPase 2
RARG: Retinoic Acid Receptor Gamma
ROS: Reactive Oxygen Species
SNP: Single Nucleotide Polymorphism
SLC22A16: Solute Carrier Family 22 Member 16
SLC28A3: Solute Carrier Family 28 Member 3
SOD2: Superoxide Dismutase 2
STMN1: Stathmin 1
SULT2B1: Sulfotransferase Family 2B Member 1
TNF: Tumor Necrosis Factor
TOP2B: DNA Topoisomerase II Beta
TP53: Tumor Protein p53
TRPC6: Transient Receptor Potential Cation Channel Subfamily C Member 6
UGT1A6: UDP Glucuronosyltransferase Family 1 Member A6
UGT2B7: UDP Glucuronosyltransferase Family 2 Member B7
