# Supplementary material for: Pharmacogenomic predictors of anthracycline-induced cardiotoxicity in breast cancer patients: a systematic review and meta-analysis
Source: Cardiooncology. 2026 Mar 3;12:49. doi: 10.1186/s40959-026-00449-3 (PMC13064243; doi:10.1186/s40959-026-00449-3)
Supplement: Supplementary file 1 — Supplementary Material 1. [file 40959_2026_449_MOESM1_ESM.docx]

**SUPPLEMENTARY DATA**

**Supplementary Table 1 (S1): PubMed Search Strategy**

| Search number | Query | Search Results  **(June 13, 2025)** |
| --- | --- | --- |
| 13 | (((#1) AND (#10)) AND (#11)) AND (#12) | 46 |
| 12 | (#8) OR (#9) | 4,20,798 |
| 11 | (#6) OR (#7) | 2,32,505 |
| 10 | (((#2) OR (#3)) OR (#4)) OR (#5) | 1,25,359 |
| 9 | (((((((((((((Cardiac Failure) OR (Heart Decompensation)) OR (Decompensation, Heart)) OR (Congestive Heart Failure)) OR (Heart Failure, Congestive)) OR (Heart Failure, Right-Sided)) OR (Heart Failure, Right Sided)) OR (Right-Sided Heart Failure)) OR (Right Sided Heart Failure)) OR (Heart Failure, Left-Sided)) OR (Heart Failure, Left Sided)) OR (Left-Sided Heart Failure)) OR (Left Sided Heart Failure)) OR (Myocardial Failure) | 3,85,782 |
| 8 | (((Cardiotoxicities) OR (Cardiac Toxicity)) OR (Cardiac Toxicities)) OR (Toxicity, Cardiac) | 42,173 |
| 7 | (((((Nucleotide Polymorphism, Single) OR (Nucleotide Polymorphisms, Single)) OR (Polymorphisms, Single Nucleotide)) OR (Single Nucleotide Polymorphisms)) OR (Single Nucleotide Polymorphism)) OR (SNPs) | 2,02,039 |
| 6 | Pharmacogenomics | 37,435 |
| 5 | (((((((((((((Dauno-Rubidomycine)) OR (Dauno Rubidomycine)) OR (Daunomycin)) OR (Rubidomycin)) OR (Rubomycin)) OR (Cerubidine)) OR (NSC-82151)) OR (NSC82151)) OR (NSC 82151)) OR (Daunorubicin Hydrochloride)) OR (Hydrochloride, Daunorubicin)) OR (Daunoblastin)) OR (Daunoblastine) | 79,374 |
| 4 | (((((((((((((((((((((((((4'-Epiadriamycin) OR (4' Epiadriamycin)) OR (4'-Epidoxorubicin)) OR (4'-Epi-DXR)) OR (4' Epi DXR)) OR (4'-Epi-Adriamycin)) OR (4' Epi Adriamycin)) OR (4'-Epi-Doxorubicin)) OR (4' Epi Doxorubicin)) OR (Ellence)) OR (Farmorubicin)) OR (Farmorubicine)) OR (Pharmorubicin)) OR (Epirubicin Hydrochloride)) OR (Hydrochloride, Epirubicin)) OR (IMI-28)) OR (IMI28)) OR (IMI 28)) OR (NSC-256942)) OR (NSC256942)) OR (NSC 256942)) OR (Epilem)) OR (EPI-cell)) OR (EPIcell)) OR (EPI cell)) OR (Farmorubicina) | 13,311 |
| 3 | (Doxorubicina Tedec)) OR (Doxorubicina Funk)) OR (Doxorubicina Ferrer Farm)) OR (Doxorubicin NC)) OR (Adrimedac)) OR (Doxorubicin Hexal)) OR (DOXO-cell)) OR (DOXO cell)) OR (Urokit Doxo-cell)) OR (Urokit Doxo cell)) OR (Myocet)) OR (Farmiblastina)) OR (Doxotec)) OR (Ribodoxo)) OR (Onkodox)) OR (Rubex)) OR (Adriamycin)) OR (Adriblastin)) OR (Adriablastine)) OR (Adriablastin)) OR (Adriblastina)) OR (Adriblastine) | 96,585 |
| 2 | Anthracycline | 91,079 |
| 1 | ((((((((((((((((((((((((((((((((((((Breast Neoplasm) OR (Neoplasm, Breast)) OR (Neoplasms, Breast)) OR (Breast Tumors)) OR (Breast Tumor)) OR (Tumor, Breast)) OR (Tumors, Breast)) OR (Breast Cancer)) OR (Cancer, Breast)) OR (Cancer of Breast)) OR (Cancer of the Breast)) OR (Malignant Neoplasm of Breast)) OR (Breast Malignant Neoplasm)) OR (Breast Malignant Neoplasms)) OR (Malignant Tumor of Breast)) OR (Breast Malignant Tumor)) OR (Breast Malignant Tumors)) OR (Mammary Cancer)) OR (Cancer, Mammary)) OR (Cancers, Mammary)) OR (Mammary Cancers)) OR (Mammary Neoplasms, Human)) OR (Human Mammary Neoplasm)) OR (Human Mammary Neoplasms)) OR (Neoplasm, Human Mammary)) OR (Neoplasms, Human Mammary)) OR (Mammary Neoplasm, Human)) OR (Breast Carcinoma)) OR (Breast Carcinomas)) OR (Carcinoma, Breast)) OR (Carcinomas, Breast)) OR (Mammary Carcinoma, Human)) OR (Carcinoma, Human Mammary)) OR (Carcinomas, Human Mammary)) OR (Human Mammary Carcinomas)) OR (Mammary Carcinomas, Human)) OR (Human Mammary Carcinoma) | 5,74,537 |

| Search no | Query | Results |
| --- | --- | --- |
| #13 | #1 AND #10 AND #11 AND #12 | 149 |
| #12 | #8 OR #9 | 897734 |
| #11 | #6 OR #7 | 306837 |
| #10 | #2 OR #3 OR #4 OR #5 | 333317 |
| #9 | 'heart failure'/exp OR 'heart failure' | 848054 |
| #8 | 'cardiotoxicity'/exp OR 'cardiotoxicity' | 64678 |
| #7 | 'single nucleotide polymorphism'/exp OR 'single nucleotide polymorphism' | 281866 |
| #6 | 'pharmacogenomics'/exp OR 'pharmacogenomics' | 31687 |
| #5 | 'daunorubicin'/exp OR 'daunorubicin' | 36048 |
| #4 | 'epirubicin'/exp OR 'epirubicin' | 36379 |
| #3 | 'doxorubicin'/exp OR 'doxorubicin' | 254584 |
| #2 | 'anthracycline antibiotic agent'/exp OR 'anthracycline antibiotic agent' | 326808 |
| #1 | 'breast tumor'/exp OR 'breast tumor' | 768670 |

**Supplementary Table 2 (S2) : Embase Search Strategy**

**Supplementary Table 3 (S3): Summary of the genetic variant associations and AIC risk reported in various genetic models with odds ratios (OR) and 95% confidence intervals (CI).**

| **S.no** | **Gene** | **SNP (rsID)** | **Genetic Model** | **Genotype Comparison** | **OR (95% CI)** | **p-value** | **References** |
| --- | --- | --- | --- | --- | --- | --- | --- |
| 1 | ABCC1 | rs148350 | Dominant (T allele) | TG *vs* GG | 8.000 (1.405-45.547) | 0.019 | Muckiene et al. 2023(13) |
|  |  |  | Dominant (T allele) | TT+TG *vs* GG | 5.333 (1.138-24.985) | 0.034 |  |
|  |  | rs3743527 | Heterozygote | CT *vs* CC | 0.995 (0.329- 3.013) | 0.993 |  |
|  |  |  | Recessive (T allele) | TT *vs* CC | 0.483 (0.051- 4.586) | 0.527 |  |
|  |  |  | Dominant (T allele) | TT + CT *vs* CC | 0.879 (0.307-2.517) | 0.81 |  |
|  |  |  | Recessive (T allele) | TT *vs* CT+ CC | 0.484 (0.053-4.425) | 0.521 |  |
|  |  | rs246221 | Homozygote | CC *vs* TT | 0.72 (0.29-1.76) | 0.468 | Vulsteke et al. 2015(28) |
|  |  |  | Heterozygote | TC *vs* TT | 1.59 (1.07-2.35) | 0.021 | Vulsteke et al. 2015(28) |
| 2 | ABCB1 | rs104564 | Heterozygote | CT *vs* TT | 0.667 (0.187-2.379) | 0.532 | Muckiene et al. 2023(13) |
|  |  |  | Homozygote | CC *vs* TT | 1.000 (0.200-5.004) | 1 |  |
|  |  |  | Dominant (C allele) | CC + CT *vs* TT | 0.732 (0.215-2.492) | 0.617 |  |
|  |  |  | Recessive (T allele) | CT + TT *vs* CC | 1.344 (0.355-5.083) | 0.663 |  |
|  |  | rs1045642 | Additive | CC=0, CT=1, TT=2 | 0.48 (0.23 –1.00) | 0.049 | Hertz et al. 2016(24) |
| 3 | CBR3 V244M | rs1056892 | Homozygote | AA *vs* GG | 0.556(0.186–1.658) | 0.292 | Vaitiekus et al. 2025(14) |
|  |  |  | Heterozygote | AG *vs* GG | 0.682(0.354–1.314 | 0.253 |  |
|  |  |  | Dominant (A allele) | AA, AG + GG | 0.645(0.368–1.132) | 0.127 |  |
|  |  |  | Recessive (A allele) | AA vs AG + GG | 0.933(0.281–3.099) | 0.91 |  |
|  |  |  | Homozygote comparison | AA *vs* GG | 2.55 (0.26, 25.17) | 0.424 | Lang et al. 2021(15) |
|  |  |  | Heterozygote | GA *vs* AA | 1.43 (0.15 to 13.26) | 0.754 |  |
|  |  |  | Additive | GG=0, GA=1, AA=2 | 2.50 (1.22–5.11) | 0.012 | Hertz et al. 2016(24) |
|  |  |  | Recessive (A allele) | AA *vs* GA+GG | 6.19 (1.94–19.76) | 0.002 |  |
|  |  |  | Allelic |  | 1.82 (0.65- 5.26) | 0.259 | Advani et al. 2023(17) |
|  |  |  | Genotype categories | AA *vs* AG *vs* GG | Not Reported | NR | Volkan-Salanci et al. 2012(34) |
| 4 | CBR1 | rs9024 | Homozygote | AA *vs* GG | 0.556(0.186–1.658) | 0.292 | Vaitiekus et al. 2025(14) |
|  |  |  | Heterozygote | AG *vs* GG | 0.682(0.354–1.314) | 0.253 |  |
|  |  |  | Dominant (A allele) | AA+ AG *vs* GG | 0.645(0.368–1.132) | 0.127 |  |
|  |  |  | Recessive (A allele) | AA *vs* AG+ GG | 0.933(0.281–3.099) | 0.91 | Vaitiekus et al. 2025(14) |
| 5 | CYBA | rs1049255 | Homozygote comparison | AA *vs* GG | 0.688(0.319–1.481) | 0.339 | Vaitiekus et al. 2025(14) |
|  |  |  | Heterozygote | AG *vs* GG | 0.500(0.257–0.973 | 0.041 |  |
|  |  |  | Dominant (A allele) | AA+AG *vs* GG | 0.571(0.346–0.944) | 0.029 |  |
|  |  |  | Recessive (A allele) | AA *vs* AG+GG | 1.266(0.490–3.273) | 0.626 |  |
|  | HFE (H63D) | rs1799945 | Allelic | G *vs* C | 3.44 (1.40–8.47) | 0.005 | Vaitiekus et al. 2021(18) |
|  |  |  | Genotype-specific | GC *vs* CC | 3.02 (1.06–8.59) | 0.038 |  |
|  |  |  | Dominant | GG + GC *vs* CC | 3.57 (1.06–8.59) | 0.014 |  |
| 6 | NCF4 | rs1883113 | Homozygote | AA *vs* GG | 0.500(0.151–1.660) | 0.258 | Vaitiekus et al. 2025(14) |
|  |  |  | Heterozygote | AG *vs* GG | 0.481(0.248–0.933) | 0.03 |  |
|  |  |  | Dominant (A allele) | AA+ AG *vs* GG | 0.486(0.272–0.867) | 0.015 |  |
|  |  |  | Recessive (A allele) | AA *vs* AG+ GG | 0.827(0.226–3.020) | 0.774 |  |
|  |  |  | Recessive (A allele) | AA *vs* AG + GG | 1.44 (0.51–4.07) | 0.6 | Hertz et al. 2016(24) |
|  |  |  | Heterozygote | AG *vs* AA | 1.47 (0.86–2.51) | 0.157 | Vulsteke et al. 2015(28) |
|  |  |  | Homozygote | GG *vs* AA | 0.92 (0.51–1.68) | 0.793 |  |
| 7 | TRPC6 | rs77679196 | Allelic |  | 12.84 (1.24- 133.2) | 0.032 | Advani et al. 2023(17) |
|  |  | rs767086724 | Allelic | A' minor allele | 0.026 | 0.126 | Norton et al. 2020(19) |
|  |  | rs57242572 | Allelic | C' minor allele | 0.013 | 0.014 |  |
|  |  |  | Allelic | T' minor allele | 0.053 | 0.031 |  |
|  |  | rs61918162 | Allelic | C' minor allele | 0.276 | 0.065 |  |
| 8 | RAC2 | rs13058338 | Additive | TT = 0, TA = 1, AA = 2. | 0.75 (0.31–1.82) | 0.38 | Hertz et al. 2016(24) |
| 9 | SLC28A3 | rs7853758 | Additive | GG = 0, GA = 1, AA = 2. | 0.55 (0.16–1.91) | 0.43 | Hertz et al. 2016(24) |
| 10 | TOP2B | rs10865801 | Additive | CC = 0, CT = 1, TT = 2. | 1.32 (0.67–2.61) | 0.47 | Hertz et al. 2016(24) |
| 11 | NADPH oxidase | rs4673 | Homozygote comparison | С/С | 0.6508 (0.3137–1.3499) | 0.2485 | Grakova et al. 2021  (31) |
|  |  |  | Heterozygote | C/T | 0.6996 (0.3629–1.3487) | 0.2861 |  |
|  |  |  | Homozygote comparison | T/T | 2.7529 (1.3066–5.8005) | 0.0077 |  |
| 12 | NOS3 | rs1799983 | Homozygote comparison | G/G | 0.5296 (0.2749–1.0203) | 0.0574 | Grakova et al. 2021(31) |
|  |  |  | Heterozygote | G/T | 1.1241 (0.5718–2.2099) | 0.7345 |  |
|  |  |  | Homozygote | T/T | 3.0585 (1.2094–7.7348) | 0.0182 |  |
| 13 | EDNRA | rs5335 | Homozygote comparison | G/G | 1.10 (0.4826–2.5341) | 0.8242 | Grakova et al. 2021(31) |
|  |  |  | Heterozygote | C/G | 0.8709 (0.4542–1.6700) | 0.6773 |  |
|  |  |  | Homozygote comparison | C/C | 1.1154 (0.5372–2.3156) | 0.8176 |  |
| 14 | PON1 | rs662 | Homozygote comparison | GG *vs* AA | 0.517 (0.05-5.321) | 0.579 | Vaitiekus et al. 2025(14) |
|  |  |  | Heterozygote | AG *vs* AA | 0.821 (0.307-2.196) | 0.694 |  |
|  |  |  | Dominant (G allele) | GG + AG *vs* AA | 0.775 (0.301–1.993) | 0.597 |  |
|  |  |  | Recessive (G allele) | GG *vs* AG+AA | 0.552(0.055–5.556) | 0.614 |  |
| 15 | SULT2B1 | rs10426377 | Homozygote | AA *vs* CC | 0.857(0.192–3.830) | 0.84 | Vaitiekus et al. 2025(14) |
|  |  |  | Heterozygote | AC *vs* CC | 1.625(0.614–4.301) | 0.328 |  |
|  |  |  | Dominant (A allele) | AA+ AC *vs* CC | 1.391(0.563–3.439) | 0.474 |  |
|  |  |  | Recessive (A allele) | AA *vs* AC+CC | 0.698(0.166–2.933) | 0.624 |  |
